# Copper Cytotoxicity: Cellular Casualties of Noncognate Coordination Chemistry

**DOI:** 10.1128/mbio.00434-22

**Published:** 2022-05-23

**Authors:** Charlotte I. Z. O’Hern, Karrera Y. Djoko

**Affiliations:** a Department of Biosciences, Durham University, Durham, United Kingdom

**Keywords:** copper, bacteria, copper homeostasis, metalloenzyme, metalloprotein, mismetalation

## Abstract

Copper (Cu) is an essential micronutrient for cells, but in excess it is cytotoxic. How Cu is cytotoxic is the subject of recent work by L. Zuily, N. Lahrach, R. Fassler, O. Genest, et al. (mBio 13:e03251-21, 2022, https://doi.org/10.1128/mbio.03251-21). Using Escherichia coli as the model cell, the work shows that anoxic cells accumulate larger amounts of Cu than do oxic cells. Accordingly, Cu is more cytotoxic under anoxic than oxic conditions. The work further shows that Cu cytotoxicity in anoxic bacteria is associated with increased intracellular protein aggregation. The mechanistic details remain as open questions, but these questions highlight that a fundamental understanding of Cu speciation and availability in cells is essential to uncover the cellular consequences of Cu cytotoxicity.

## Abstract (precis)

Copper (Cu) is an essential micronutrient for cells, but in excess it is cytotoxic. How Cu is cytotoxic is the subject of recent work by L. Zuily, N.

## COMMENTARY

### Why is copper (Cu) cytotoxic?

Fundamentally, Cu cytotoxicity is a problem with metal speciation. Cu is a competitive metal for binding to proteins and other biomolecules (1). A Cu ion that enters a cell will fill any available Cu-binding site in that cell. Nutrient Cu will bind to cognate, high-affinity, specific sites—for example, in cuproenzymes, where Cu plays functional roles. Once these nutritional Cu sites are filled, excess intracellular Cu will bind to cognate, high-affinity sites in Cu-sensing transcriptional sensors. In turn, these sensors upregulate the expression of Cu homeostasis genes, whose protein products secure, sequester, and expel any surplus Cu from the cell. If this homeostatic capacity is impaired or overwhelmed, the excess Cu will bind aberrantly to noncognate, low-affinity, nonspecific sites. Such messy metal speciation, termed “mismetalation,” is what ultimately leads to cell damage and death.

Likely target sites for mismetalation by Cu comprise the metal-binding sites in other metalloproteins and metalloenzymes. Cu can become incorrectly inserted into these sites during the metalloprotein maturation process. Alternatively, Cu can displace the cognate metals from mature metalloproteins. The displaced metals are released into the cellular milieu, which can lead to a cascade of subsequent mismetalation events. Other target sites include proteins and enzymes that do not normally bind metals but nonetheless contain solvent-accessible, potential Cu-binding ligands, such as the side chains of Met, His, or Cys. Here, Cu can change the local protein conformation. Cu can also promote oxidation of Cys thiols or formation of incorrect disulfide bonds. Regardless of the specific mechanism, mismetalation by Cu universally leads to a loss of protein or enzyme function and, if uncorrected, subsequent cell damage and death.

### Why is Cu more cytotoxic under anoxic conditions?

What happens to a cell when there is excess intracellular Cu depends on Cu speciation: i.e., where Cu binds. Precisely where Cu binds depends on target availability (what possible Cu-binding sites are present in the cell) and Cu availability (how much Cu is needed for this metal to bind to a site given its position in the binding hierarchy of all possible Cu sites) in the cell. These will vary according to the identity of the cell and, importantly, the cellular context. The latter includes the amounts of extracellular Cu, the redox form of Cu, the presence of molecules that can and cannot chelate Cu in the extracellular milieu, the length of Cu exposure, the oxygenation status, and the supply of nutrients, which can all influence the final cellular outcome.

For example, Cu is frequently observed to be more cytotoxic to bacteria under anoxic culture conditions (compared with oxic conditions) (2–5). When exposed to the same levels (total concentrations) of extracellular Cu, bacteria that are cultured under anoxic conditions can become inhibited and/or die, while the same bacteria that are cultured under oxic conditions survive. When exposed to the same levels of extracellular Cu, anoxic bacteria are also known to accumulate more intracellular (or at least cell-associated) Cu than do oxic bacteria (2, 4). Why this occurs is unknown, but it can be any combination of (i) an increase in extracellular Cu availability, (ii) an increase in nonspecific Cu uptake by the bacterial cell, (iii) an increase in intracellular Cu sequestration and buffering inside the cell, and/or (iv) a decrease in Cu efflux from the cell. In any case, the model is relatively simple: more intracellular Cu leads to more cytotoxicity.

Yet, the work by Zuily and colleagues using Escherichia coli shows that bacterial death under oxic conditions is associated with *lower* levels of intracellular (or cell-associated) Cu (approximately half of those in anoxic bacteria) (6). Thus, although *extracellular* Cu is more cytotoxic under *anoxic* conditions, *intracellular* Cu appears to be more cytotoxic under *oxic* conditions.

This key observation highlights the difficulty in defining and measuring Cu speciation and availability. Cu may mismetalate different target sites inside oxic and anoxic bacterial cells as a result of fundamental differences between the oxic and anoxic cellular proteomes. Alternatively, Cu may mismetalate the same target sites, but these sites may be essential to the bacterial cell only under oxic, but not anoxic, conditions. Or the same target sites may be present: these sites may be essential under both oxic and anoxic conditions, but intracellular Cu availability may differ. Intracellular Cu *levels* may be lower in oxic bacteria, but intracellular Cu *availability* may be higher, leading to increased Cu cytotoxicity. The model is, apparently, not so simple after all.

### Cu-induced protein aggregation—Cu speciation run amok?

The work by Zuily and colleagues further shows that high levels of intracellular (or cell-associated) Cu, achieved only in anoxically grown bacteria, correlate with a buildup of intracellular protein aggregates (6). The work thus provides *in vivo* support for *in vitro* studies that demonstrate aggregation of purified proteins in the presence of added Cu ions (7–11).

**FIG 1:**
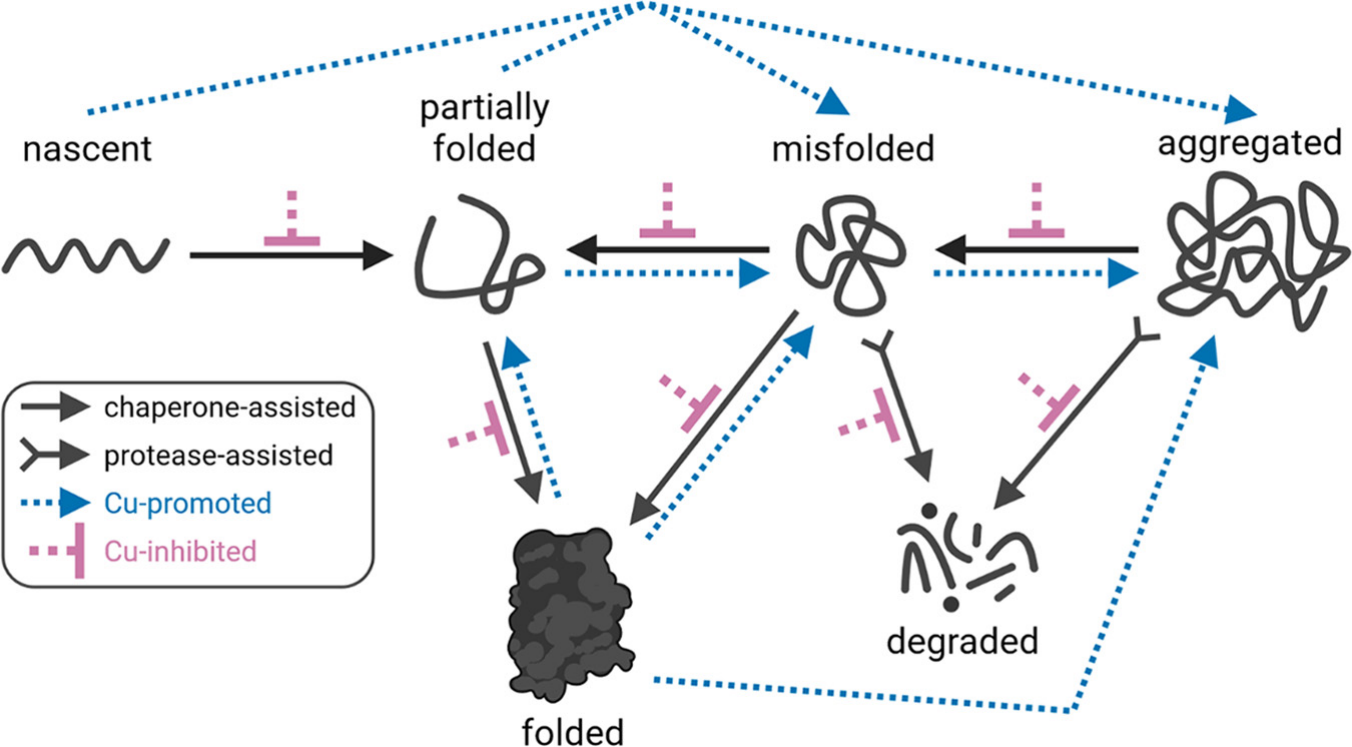
Potential links between Cu cytotoxicity and protein aggregation in cells. Cu may promote protein unfolding, misfolding, and/or aggregation. Alternatively, Cu may inhibit chaperone-assisted protein folding pathways or protease-assisted protein degradation pathways, either directly by enzyme inactivation or indirectly by depletion of cellular ATP supply. (Created using BioRender.com.)

In the simplest model, excess intracellular Cu [predominantly Cu(I) in anoxic bacteria] mismetalates multiple protein targets, as described earlier, and promotes their aggregation. Indeed, proteins that are aggregated *in vitro* in response to Cu(I) treatment do include multiple metalloproteins and proteins whose sequences are relatively enriched in His and Cys compared with those that are aggregated in response to a general stress such as heat shock (6). It will be important to determine whether these proteins are also aggregated *in vivo* and which proteins, if any, are preferentially aggregated. In any case, one key question immediately follows: where does the excess Cu bind (Fig. 1)? Does Cu bind to nascent polypeptides as they emerge from ribosomes, to partially folded intermediates, or to mature proteins? The answer may differ for different protein targets in response to different amounts of Cu, illustrating the challenge in understanding Cu speciation and availability in cells.

Proteins can aggregate, even in healthy cells, as a result of not only errors in their folding, but also in their synthesis, trafficking, or degradation (12). Housekeeping machineries typically keep these aggregates in check. Folding chaperones can solubilize and subsequently refold these aggregates into their native conformations. Alternatively, proteases can degrade and clear these aggregates entirely. An increase in the cellular concentrations of protein aggregates will sequester these folding chaperones and proteases, and transcriptionally increase the production of more chaperones and proteases (13). Indeed, the work by Zuily and colleagues shows that high levels of intracellular (or cell-associated) Cu correlate with increased levels of *dnaK* mRNA (6), which encodes the molecular folding chaperone DnaK (heat shock protein Hsp70). However, a buildup of protein aggregates in these Cu-treated bacteria clearly signals that these housekeeping machineries, even if there are more of them, are either overwhelmed or impaired.

What is the sequence of events here (Fig. 1)? Does excess intracellular Cu promote widespread protein aggregation, which overwhelms the housekeeping machineries? Or does excess intracellular Cu impair the housekeeping machineries, which leads to the buildup of widespread protein aggregation? Cu may inactivate these machineries directly by mismetalating the folding chaperones and proteases or indirectly by mismetalating an upstream molecule. For example, inhibition of GAPDH (glyceraldehyde-3-phosphate dehydrogenase) by Cu (14, 15) can lead to a global depletion in cellular ATP (especially under anoxic conditions in which oxidative phosphorylation does not occur) and subsequently stall ATP-dependent folding chaperone or protease activity. These are, once again, questions about Cu speciation. Where does the excess Cu bind?

### Protein aggregation—cytotoxic or cytoprotective?

Protein aggregation is considered cytotoxic because it essentially sequesters proteins and enzymes from the cellular milieu and stops their function. However, protein aggregation in bacteria has also been implicated in cytoprotection (16). In response to antibiotic stress, bacterial cells can enter a metabolically dormant state that is associated with increased protein aggregation. Dormant bacterial cells can better tolerate antibiotic exposure, and resuscitated cells can resume proteostasis. Is this also the case for Cu stress? Here, it is tempting to speculate that the aggregates trap Cu, lower overall Cu availability inside Cu-stressed cells, and prevent further cellular damage.

Given the established link between Cu cytotoxicity and the spread of antibiotic resistance, the increased use of metallic Cu surfaces to promote hygiene in clinical care settings, and the key role of Cu as an antimicrobial effector in host immune systems, the work by Zuily and colleagues certainly prompts multiple new, fundamental questions with important real-world implications.
